# Local Resection by Combined Laparoendoscopic Surgery for Duodenal Gastrointestinal Stromal Tumor

**DOI:** 10.1155/2011/645609

**Published:** 2011-07-27

**Authors:** Motohiko Kato, Kiyokazu Nakajima, Toshirou Nishida, Makoto Yamasaki, Tsutomu Nishida, Shusaku Tsutsui, Hideharu Ogiyama, Shunsuke Yamamoto, Takuya Yamada, Masaki Mori, Yuichiro Doki, Norio Hayashi

**Affiliations:** ^1^Department of Gastroenterology and Hepatology, Osaka University Graduate School of Medicine, Suita, Osaka 565-087, Japan; ^2^Department of Gastroenterological Surgery, Osaka University Graduate School of Medicine, 2-2, E-2, Yamadaoka, Suita, Osaka 565-0871, Japan; ^3^Department of Gastroenterological Surgery, Osaka Police Hospital, Japan; ^4^Kansai-Rosai Hospital, Amagasaki, Japan

## Abstract

Combined laparoendoscopic surgery is a novel surgical method which consists of both endoscopic surgery from inside the gastrointestinal tract and laparoscopic surgery from the outside. We report a case of duodenal GIST, in which combined laparoendoscopic local resection was attempted. The lesion was resected endoscopically using endoscopic submucosal dissection technique under laparoscopic assistance. Laparoscope was used for originating the orientation of the tumor, intra-operative EUS, and monitoring serosal injury from the peritoneal cavity. Postoperative hemorrhage occurred; however, precise orientation of the lesion helped us to manage the patient with minimal invasive reoperation. And thus, the bowel integrity was completely preserved, by avoiding segmental duodenal resection and pancreaticoduodenectomy. This novel, less invasive surgical procedure may become an attractive option for the lesions originating in the anatomically challenging portion of the GI tract for endoscopic or laparoscopic surgery alone.

## 1. Introduction

Even with recent advantage of endoscopic and laparoscopic technology, duodenum is still a challenging organ for minimal invasive surgery due to its anatomical properties. Recently there have been various reports concerning “combined laparo-endoscopic surgery,” which consist of both endoscopic surgery from inside the gastrointestinal tract and laparoscopic surgery from the outside. Here, we report a case of duodenal SMT applied this novel surgical procedure. 

## 2. Case Report

**Figure 1:**
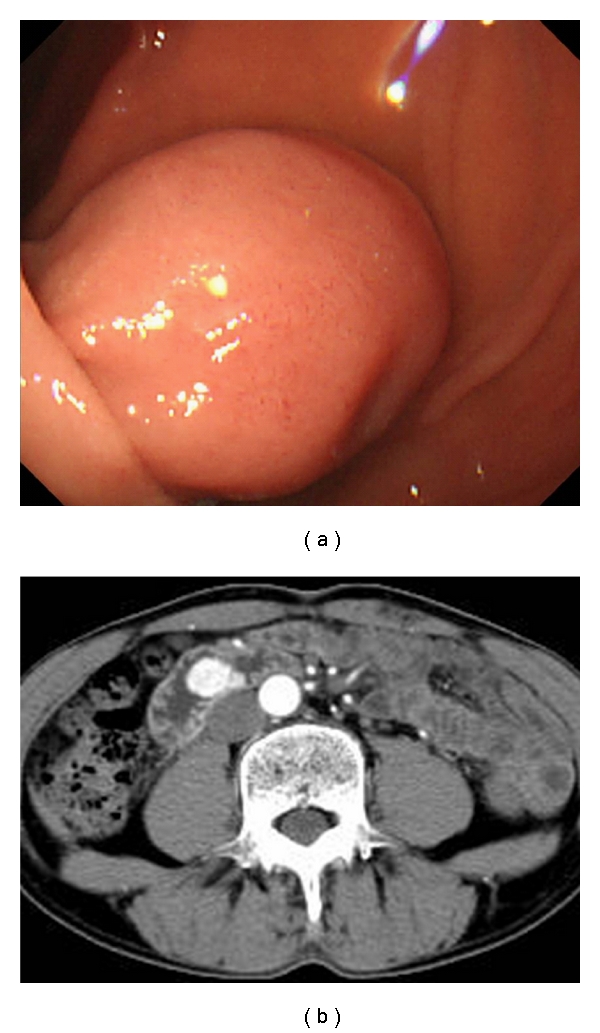
(a) Endoscopic appearance revealed a 20-mm diameter submucosal tumor with a smooth surface in the third portion of the duodenum. (b) Preoperative endoscopic ultrasonography (12 MHz miniature probe). Arrowheads indicate that muscle layer was preserved beneath the tumor without an extramural component.

A male patient in his 60s visited our hospital because he was diagnosed with a submucosal tumor (SMT) of the duodenum that had progressed in size during 3-month follow up. esophagogastroduodenoscopy revealed a 20-mm diameter SMT located in the third portion of the duodenum (Figure 1(a)). CT scan revealed hypervascular tumor existing in the third portion of duodenum (Figure 1(b)). An 18 F-fluorodeoxyglucose-positron emission tomography-computed tomography (FDG-PET-CT) scan showed a homogenous submucosal lesion without lymph node swelling nor distant metastasis. The initial surgical consultation indicated segmental duodenal resection with Roux-en Y reconstruction, or in a worst case scenario, pancreaticoduodenectomy. The lesion, however, was considered as a low-risk GIST according to the recent NCCN sarcoma guidelines [1]. The surgical team, therefore, offered the endoscopic resection under laparoscopic assistance as less invasive alternative to segmental duodenectomy and pancreaticoduodenectomy.

**Figure 2:**
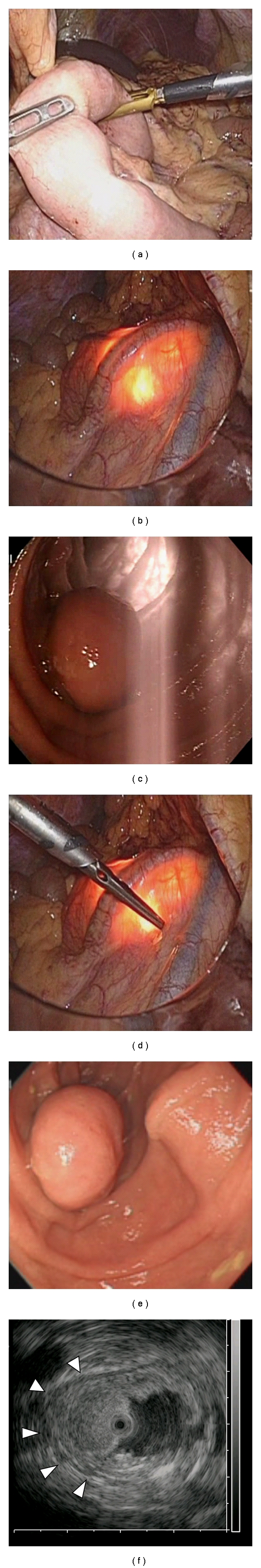
(a) Proximal jejunum was clamped using intestinal forceps to avoid distention of the distal bowel by the laparoscope. (b, c) Laparoscopic and endoscopic view. Transmitted light of both flexible endoscopy and laparoscopy could be seen through the duodenal wall. (d, e) Identification of the tumor location by poking the duodenal wall.

**Figure 3:**
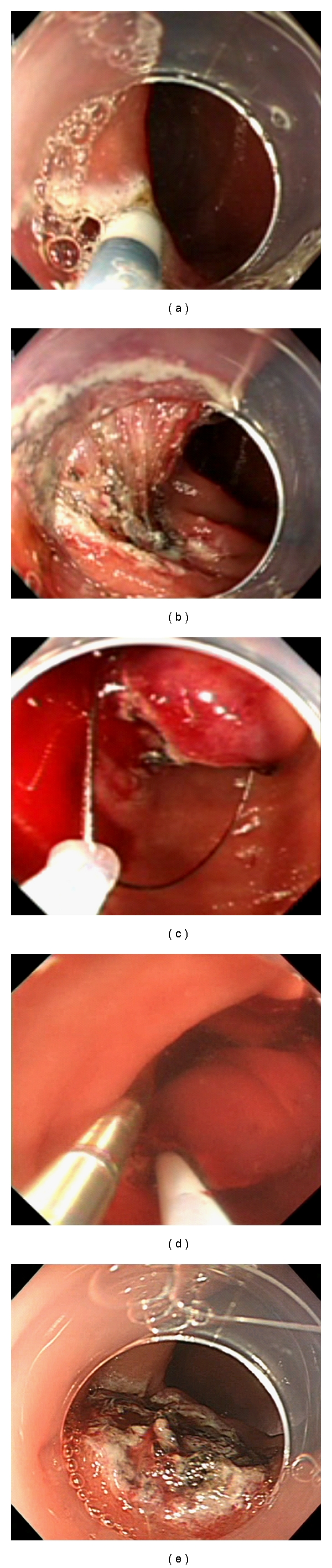
(a) Mucosal incision and following submucosal dissection was performed by flush knife. (b) The tumor was got well mobilized, when the incision was made almost circumferentially except for the anal side of the tumor. (c) The root of the lesion was grasped with an electric snare. (d) EUS was performed during grasping the lesion with an electric snare with 2-channel endoscope to confirm resectability. (e) Postresected ulcer.

 The procedure was performed at our surgical unit under general anesthesia. After establishment of standard CO_2_ pneumoperitoneum, three surgical ports were placed at the umbilicus, right, and left midabdomen, respectively. The peritoneal cavity was explored laparoscopically, and the proximal jejunum was gently clamped (Figure 2(a)). A flexible endoscope (GIF-H260Z, Olympus Medical Systems Co. Ltd, Tokyo, Japan) was inserted perorally with a CO_2_ feeding system (UCR, Olympus Medical Systems Co. Ltd, Tokyo, Japan). The endoscope was then advanced into duodenum, and we confirmed the lesion located at the third portion of duodenum with transmitted light from flexible endoscopy (Figures 2(b) and 2(c)). Subsequently, we confirmed that the lesion existed in the posterior wall by picking the anterior wall of duodenum (Figures 2(d) and 2(e)). After filling the cavity of duodenum with water, intraoperative endoscopic ultrasonography (EUS) was performed. EUS was performed using a radial-scanning, 20-MHz catheter probe (UM3D-DP20-25R, Olympus, Tokyo, Japan). The lesion revealed protruding toward the lumen without an extramural component (Figure 2(f)). The lesion was elevated by injecting physiological saline with epinephrine into the submucosal layer in a standard fashion. A mucosal incision was made around the tumor, and the submucosal layer was dissected just below the tumor with a flush knife (Fujinon Toshiba ES Systems Co. Ltd, Tokyo, Japan) (Figure 3(a)). An ICC200 electrosurgical generator (ERBE, Tubingen, Germany) was used. Because GISTs usually arise from the muscularis propria, we planned a full-thickness resection with laparoscopic enclosure. When the incision was made almost circumferentially except for the anal side of the tumor (Figure 3(b)), the tumor became well mobilized and was found to be located mainly in the submucosal layer using concurrent EUS. Therefore, we decided to resect the lesion by snarectomy alone. Repeated EUS was performed after the lesion was grasped by its roots using an electric snare with a 2-channel endoscope (GIF-2T240; Olympus Medical Systems Co. Ltd, Tokyo, Japan), which revealed that the muscle layer was not involved under the snare, and the tumor was successfully resected (Figures 3(c) and 3(d)). The specimen was isolated and delivered perorally, and an intraoperative frozen section confirmed a free vertical margin pathologically. At the conclusion of the procedure, the mucosal defect was carefully inspected and left opened, since no major submucosal vessels were observed (Figure 3(e)). The duration of the procedure was 200 min, and blood loss was negligible.

 The patient was sent back to the ward in a stable condition, but gastrointestinal bleeding occurred several hours after the procedure. Emergent endoscopy revealed a mass spurting from the resected ulcer bed, and the hemostasis was obtained with high-frequency forceps. The patient, however, showed rebleeding on the postoperative day 1. Endoscopic hemostasis was again attempted, but the bleeder could not be identified due to massive coagula in the duodenum. We decided it is impossible to obtain hemostasis endoscopically. Since we had known postresected ulcer existed at posterior wall of the third portion of the duodenum, we made minilaparotomy and small duodenotomy of the anterior wall, and the bleeder was identified on the mucosal defect. The hemostasis was immediately obtained by suturing the bleeder with 3-0 absorbable suture. The mucosal defect was closed, and the duodenoplasty was performed in “Heineke-Mikulicz” fashion. The patient showed rapid and uneventful recovery afterwards.

**Figure 4:**
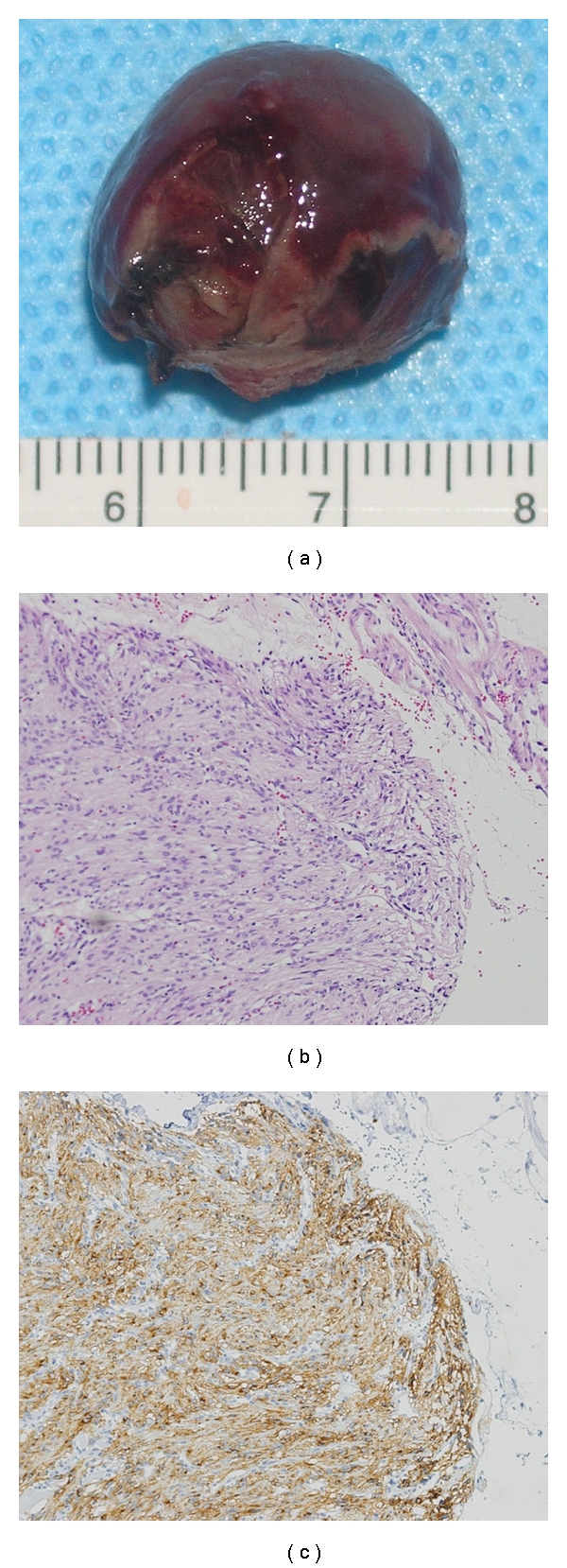
(a) Macroscopic view. Resected tumor was 18∗15∗15 mm in diameter without injury of pseudocapsule. (b) HE staining revealed that spindle cells were proliferating in the submucosal layer. Mitosis was seen less than three cells/50 HPF. (c) The majority of the tumor cell was positive for immunostaining of KIT.

 The tumor showed spindle cells that stained positive for KIT (CD117) (Figures 4(b) and 4(c)). Fewer than three mitotic cells were observed per 50 high-power fields. The final diagnosis was duodenal GIST with very low risk. At the 8-month follow-up, he remained well without any gastrointestinal symptoms nor tumor recurrence.

## 3. Discussion

Even with recent development of gene targeting therapies, surgical resection remains to be only considered to be curative. Because GIST rarely involves the lymph nodes [2], it can be treated by local or segmental resection alone. The procedure can be performed laparoscopically when the lesion is located in the stomach [3]. Most surgeons, however, are reluctant to perform laparoscopic surgery for duodenal GISTs, since local duodenal resection is technically difficult due to its retroperitoneal location and close proximity to the papilla of Vater. In addition, postoperative stenosis frequently occurs in the narrow lumen of the duodenum, especially in cases with lesions protruding toward the lumen. From these reasons, the optimal surgical procedure for duodenal GIST remains unclear [4, 5].

 Combined laparo-endoscopic surgery is a novel surgical method which consist of both endoscopic surgery from inside the gastrointestinal tract and laparoscopic surgery from the outside [6]. Intraoperative flexible endoscopy is used to facilitate localization of the lesion [6], make the incision around the lesion [7], and even resect the lesion by applying an endoscopic resection or ESD technique [6–8]. Laparoscopy is used to locate the lesion extraluminally and to monitor the endoscopic resection process. In case of deep thermal injury and/or incidental perforation, laparoscopic repair with suturing can be immediately applied. The intestinal integrity has thus been preserved with this technique. The technique has been reported to be promising, however, has limitedly been performed for selected cases with gastric GISTs. With the advantage of endoscopic submucosal dissection (ESD), it becomes to be indicated for even duodenal mucosal cancer [9]. Therefore, we applied this method for duodenal SMT with laparoscopic assistance.

 The combined laparo-endoscopic surgical method was indicated for our patient because the lesion was difficult to approach by laparoscopy alone. Prior to surgery, we considered a full-thickness resection with a laparoscopic enclosure, but the tumor became well mobilized when the most submucosal dissection was completed. Intraoperative EUS was useful to avoid grasping the muscle layer with the electric snare. Therefore, the tumor was successfully resected by endoscopic surgery alone. Moreover, detailed orientation of the lesion enabled less invasive surgery to control postoperative bleeding. The etiology remains uncertain; however, unclosed duodenal mucosal defect might be responsible. Next generation endoscopic-suturing devices, or “NOTES” closing devices, might facilitate such closure in the near future [10].

 In summary, a duodenal GIST was successfully removed with combined laparo-endoscopic surgery. The patient eventually required laparotomy for postoperative bleeding; however, invasive procedures such as segmental duodenal resection and pancreaticoduodenectomy were avoided, and his bowel integrity was completely preserved. This novel, less invasive surgical procedure may become an attractive option in the treatment of SMTs existing at the location where it takes technical hurdle for laparoscopic partial resection, such as duodenum.
